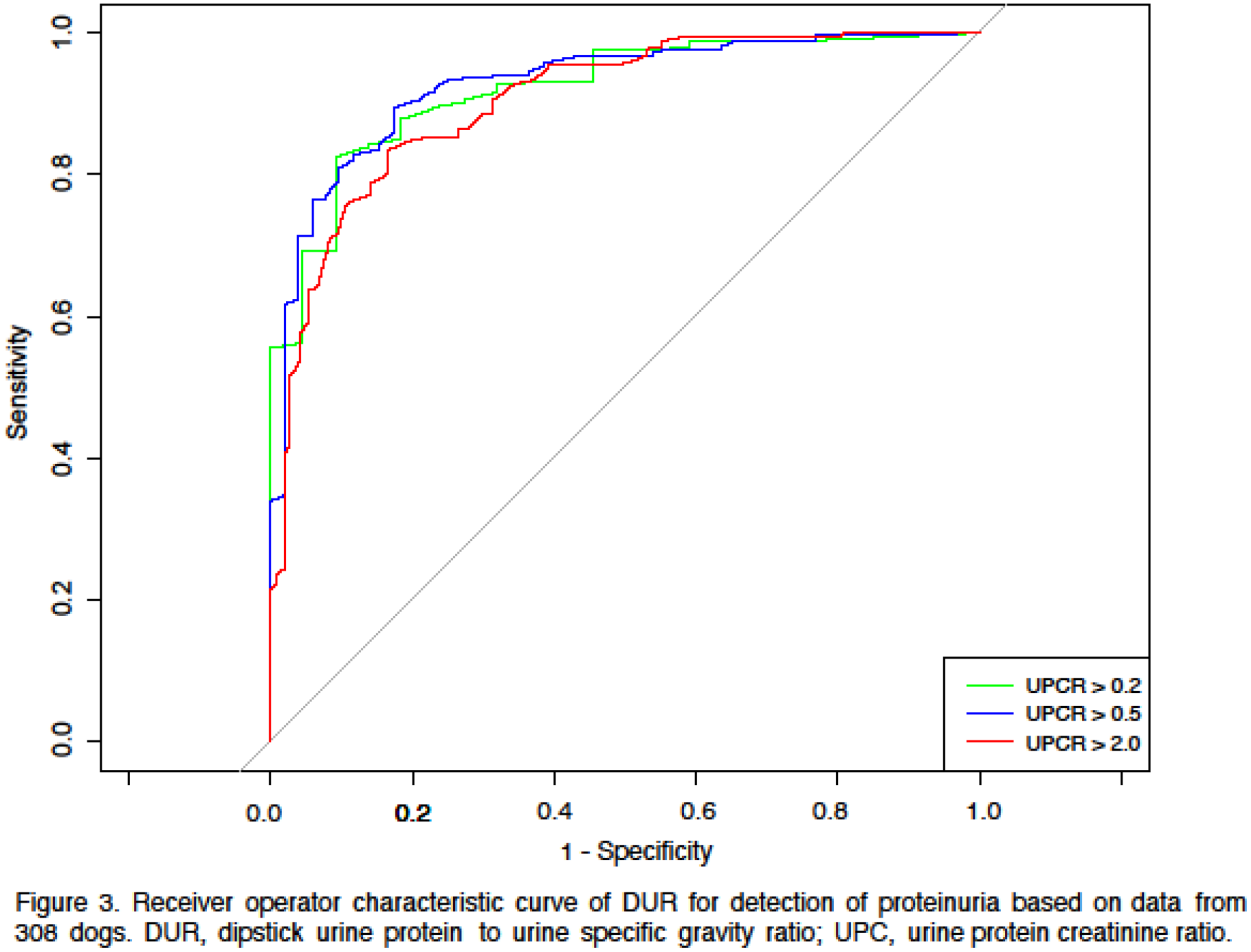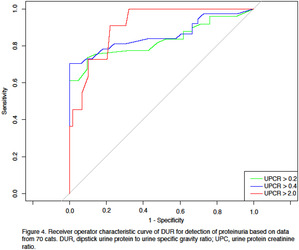# Erratum for “Evaluation of a urine dipstick protein to urine specific gravity ratio for the detection of proteinuria in dogs and cats”

**DOI:** 10.1111/jvim.17263

**Published:** 2024-12-11

Barchilon M , Perez‐Nieves N , Palerme J‐S . Evaluation of a urine dipstick protein to urine specific gravity ratio for the detection of proteinuria in dogs and cats. J Vet Intern Med. 2024;38(2):1060‐1067. doi:10.1111/jvim.17001.38305084 PMC10937511

In the article cited above, the authors have determined that the X axis is mislabeled in Figures 3 and 4. The corrected versions are shown below.

The authors regret this error.